# Prognostic Significance of Cofilin Isoforms in Patients With Pancreatic Ductal Adenocarcinoma

**DOI:** 10.3389/pore.2021.1609821

**Published:** 2021-05-10

**Authors:** Shajedul Islam, Takao Kitagawa, Yasuhiro Kuramitsu

**Affiliations:** Advanced Research Promotion Center, Health Sciences University of Hokkaido, Hokkaido, Japan

**Keywords:** biomarker, pancreatic ductal adenocarcinoma, cofilin, proteomics, kaplan-meier

**FIGURE 1:**
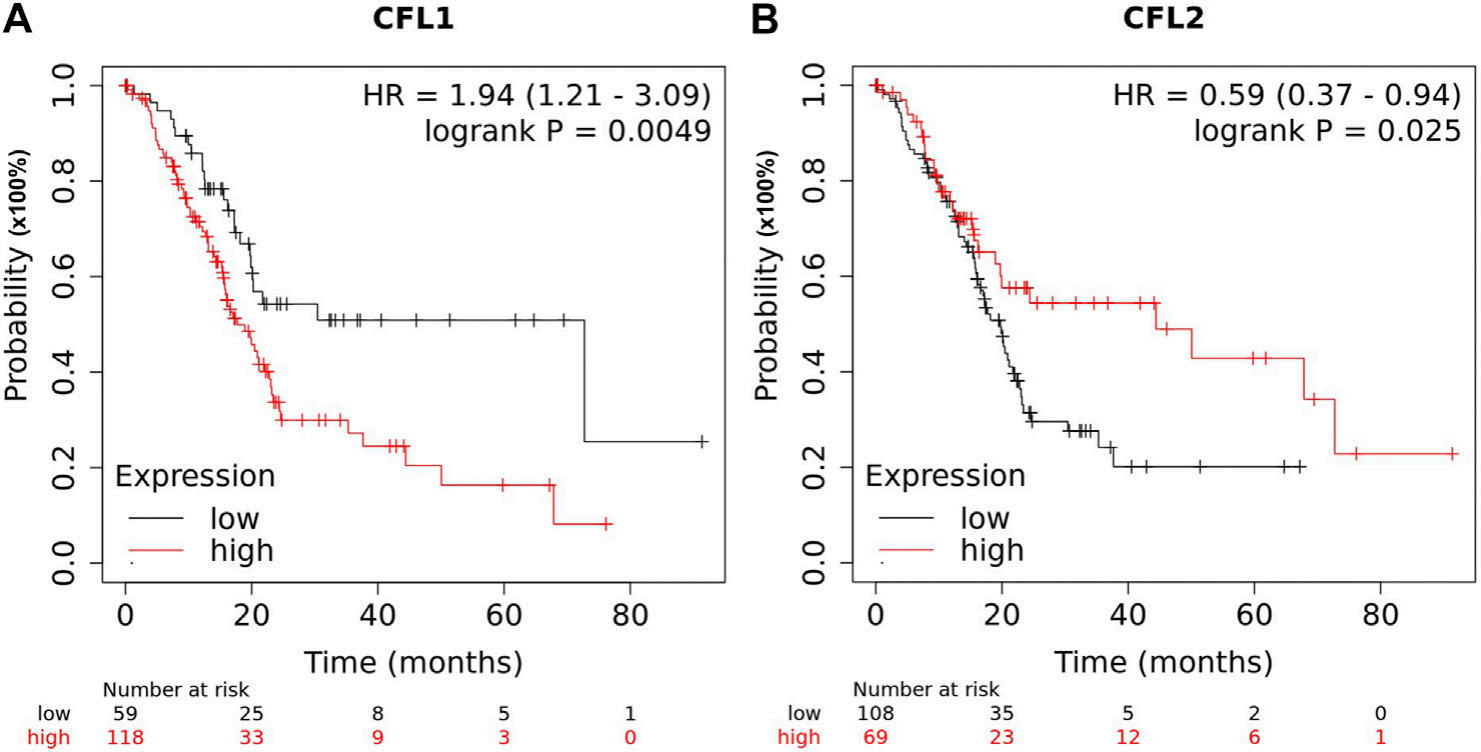
Kapan-Meier survival analysis of cofilin-1 and cofilin-2 expression in pancreatic ductal adenocarcinoma. **(A)** The survival analysis of CFL1 (cofilin-1) was performed by using the Kaplan-Meier Plotter platform. The survival plot compared a high-risk group (in red) and a low-risk group (in black) in PDAC tissues. **(B)** The survival analysis of CFL2 (cofilin-2) was performed by using the Kaplan-Meier Plotter platform. The survival plot compared a high-risk group (in red) and a low-risk group (in black) in PDAC tissues. *p* < 0.05 were regarded as statistically significant.

To the Editor,

Proteomics is a technique for analyzing protein expression at the cellular level. Proteomic technology that combines two-dimensional gel electrophoresis (2-DE) and liquid chromatography-tandem mass spectrometry (LC-MS/MS) has a high throughput and accuracy [1]. This technique is useful for comprehensively analyzing human disease proteomes and has been widely applied to detect biomarkers and molecular targets in different cancers. We previously performed differential display analysis by using 2-DE and LC-MS/MS between pancreatic cancerous and adjacent non-cancerous tissue samples [2]. One of the numerous spots that showed stronger intensity in cancerous than non-cancerous tissues was identified as non-muscle cofilin (cofilin-1). We further validated the expression of cofilin-1 by Western blot analysis. We found that cancerous tissues showed significantly higher levels of cofilin-1 than non-cancerous tissues. Meanwhile, muscle cofilin (cofilin-2), an isoform of cofilin, has also been examined in pancreatic cancerous tissue samples. Intriguingly, we observed that cancerous tissues exhibited significantly lower levels of cofilin-2 than non-cancerous tissue samples. This was the first time that cofilin isoforms were differentially expressed in pancreatic cancerous tissues, implying that they could be involved clinicopathologically in patients with PDAC. Therefore, an online bioinformatics tool (https://kmplot.com/analysis/) has been used to clarify the prognostic significance of cofilin isoforms in PDAC [3]. Using the Kaplan-Meier survival plot, we found a high level of cofilin-1 expression is inversely linked to patient survival. In contrast, high cofilin-2 is positively associated with prolonged patient survival (Figure 1). It is worth mentioning that the results of Kaplan-Meier survival plots were similar to our previous data, which showed that cofilin-1 and cofilin-2 were opposingly expressed in pancreatic cancer tissues. These observations incite interest in cofilin and focus on the relationship between their isoforms and PDAC progression. Together our previous report and the current analysis imply that cofilin isoforms may represent novel potential prognostic biomarkers in patients with PDAC. Further studies are warranted to explore the underlying mechanisms by which cofilin isoforms are involved in patients with PDAC.
